# Recognition of Grasping Patterns Using Deep Learning for Human–Robot Collaboration

**DOI:** 10.3390/s23218989

**Published:** 2023-11-05

**Authors:** Pedro Amaral, Filipe Silva, Vítor Santos

**Affiliations:** 1 Department of Electronics, Telecommunications and Informatics (DETI), Institute of Electronics and Informatics Engineering of Aveiro (IEETA), University of Aveiro, 3810-193 Aveiro, Portugal; pedro.amaral@ua.pt; 2 Department of Mechanical Engineering (DEM), Institute of Electronics and Informatics Engineering of Aveiro (IEETA), University of Aveiro, 3810-193 Aveiro, Portugal; vitor@ua.pt

**Keywords:** collaborative robotics, object recognition, hand–object interaction, grasping posture, keypoints classification

## Abstract

Recent advances in the field of collaborative robotics aim to endow industrial robots with prediction and anticipation abilities. In many shared tasks, the robot’s ability to accurately perceive and recognize the objects being manipulated by the human operator is crucial to make predictions about the operator’s intentions. In this context, this paper proposes a novel learning-based framework to enable an assistive robot to recognize the object grasped by the human operator based on the pattern of the hand and finger joints. The framework combines the strengths of the commonly available software MediaPipe in detecting hand landmarks in an RGB image with a deep multi-class classifier that predicts the manipulated object from the extracted keypoints. This study focuses on the comparison between two deep architectures, a convolutional neural network and a transformer, in terms of prediction accuracy, precision, recall and F1-score. We test the performance of the recognition system on a new dataset collected with different users and in different sessions. The results demonstrate the effectiveness of the proposed methods, while providing valuable insights into the factors that limit the generalization ability of the models.

## 1. Introduction

Human–robot collaboration (HRC) is a research topic becoming increasingly important in modern industry, driven by the need to enhance productivity, efficiency and safety in work environments [1,2,3,4,5,6]. The combination of human skills and robotic capabilities provides significant potential to improve the execution of complex and repetitive tasks. However, the effective synchronization of actions and seamless communication between partners are open challenges that need to be further addressed [7,8,9]. In recent years, there has been a remarkable trend toward endowing collaborative robots with cognitive abilities, transforming them from simple automated machines into intelligent and adaptable collaborators. This shift is driven by the increasing demand for robots that can work alongside humans, understand their intentions and actively contribute to complex tasks in dynamic environments. Collaborative cognition encompasses a range of essential abilities in order to enable robots to learn, predict and anticipate human actions [6,10,11].

In collaborative scenarios, assistive robots are designed to work alongside humans in assembly processes or maintenance operations, providing timely support in order to enhance the overall efficiency of the task. Robots can assist the human worker by delivering a component, tool or part, by holding a part while the operator works on it or by autonomously performing a specific sub-task. In any case, the ability of an assistive robot to anticipate the upcoming needs of a human operator plays a pivotal role in supporting efficient teamwork. By anticipating human intentions, actions and needs, robots can proactively assist or complement human tasks, providing timely support and improving overall efficiency [12,13,14,15].

Unlike previous approaches [13,16,17,18], our work focuses on the robot’s ability to accurately perceive and recognize the object being manipulated by the human operator as a key element to make predictions about its needs. Knowing the object in the user’s hand provides valuable contextual information, revealing both current activity and future intention. A further detail that enforces the novelty of the approach is that the object is not detected directly by its shape, size or color because, while being handled, occlusions and partial views may compromise the actual perception. Instead, we propose to infer which object is being handled (from a list of potential objects) by the grasping pattern of the hand and fingers of the operator.

The proposed concept is generic and it can be used in many human–robot interaction scenarios. The robot can use this information to adjust its posture accordingly and/or to provide relevant assistance. For example, suppose a robot is collaborating with a worker on a factory assembly line. The robot observes the worker’s action and realizes he/she just picked up a specific object. Based on object recognition, the robot can adjust its own position or prepare the required tool to assist the worker in the assembly process. In this way, the robot anticipates the human’s action and streamlines the workflow. This example illustrates our view of how anticipation can be applied in HRC scenarios to enhance interaction and overall efficiency: the robot understands the user’s needs by recognizing the object being grasped or manipulated.

This paper focuses on a novel framework for object recognition from grasping postures that combines two learning-based models. First, the system tracks the hand manipulating an object using the MediaPipe software created by Google (release 0.10.0 sourced from the official MediaPipe repository https://github.com/google/mediapipe, accessed on 31 October 2023) that extracts 21 landmarks from an input image [19]. Second, a multi-class classifier is trained to discriminate between four objects, taking the detected keypoints as input. The primary objective is to offer a proof-of-concept that showcases both the promise of the approach and the inherent limitations of the problem. The proposed solution is tailored to a specific application context in which a collaborative robot assists a human operator in industrial settings.

According to this focus, we conducted our research under conditions that reflect the practical challenges involved. As a result, we worked with a limited set of four distinct objects aligned with the practical needs of the target application domain. Furthermore, we engaged three users to participate in our study, reflecting the intended audience of non-experts who may be tasked with interacting with the system. The choice of a small number of users was driven by the goal of developing a solution that can be easily implemented by individuals without specialized training in machine learning or computer vision. While it may be tempting to expand the scope of users and objects in a controlled laboratory setting, the focused conditions under which this study was conducted better reflect the challenges and opportunities that our system seeks to address in industrial scenarios.

Another major challenge is the lack of labelled training data, as well as the time and costs of generating them. In this context, this study contributes with an easy-to-use data acquisition system integrated into the workflow of the proposed framework. The acquisition system was designed to facilitate the fast generation of training datasets, accommodating the inclusion of new users and/or additional acquisition sessions. This enhances the practicality and scalability of the approach, while positioning the work as a valuable tool for addressing the dynamic needs of such applications.

In summary, our main contributions are the following:

1. We propose a novel object recognition system that combines the AI-based MediaPipe software for extracting the hand keypoints with a deep classifier for recognizing the category of the object held by the user’s hand. To the best of our knowledge, this is a unique computational perspective to recognize the grasped objects for application in the context of intention prediction in collaborative tasks.
2. We perform extensive experiments on a new annotated dataset to demonstrate the potential and limitations of the proposed approach. In particular, we analyze the generalization performance and/or model failure both across different trials for the same user and across multiple users.

The remainder of this paper is organized as follows. Section 2 provides an overview of the existing literature and research in the field of interest, contextualizing our work within the current state of the art. Section 3 presents the core components of our proposed approach, including the data acquisition process, the preprocessing steps applied to the collected data and details of the neural network architectures implemented as multi-class classifiers. Section 4 describes the experiments carried out and it discusses the results obtained, shedding light on the potential and limitations of the proposed approach for object recognition. Finally, Section 5 outlines the main conclusions of this study and and it highlights potential avenues for future research.

## 2. Related Work

The idea underlying this study is to explore the possibility of establishing a relationship between the object grasped by the human operator and his current needs in collaborative scenarios. The immediate conceptual roots can be traced to the Activity Theory [20] whose main concept is “activity” defined as a purposeful and developing interaction between actors (“subjects”) and the world (“objects”). This framework has established itself as a key concept for research in human–computer interaction (HCI) and interaction design.

This section provides an overview of existing literature and research relevant to our study. In particular, the recognition of the object grasped by the user is the central block when it comes to achieving the main purpose. It is organized into two subsections—Section 2.1 and Section 2.2—to help categorize and differentiate methods that primarily focus on capturing and analyzing data related to the object itself from those that explicitly use data from the human hand to infer the object being grasped.

### 2.1. Object Sensing

Approaches within the “object sensing” category leverage visual information extracted from images or videos of the objects the user is interacting with, by using techniques from computer vision and machine learning to discern object identities based on their visual attributes. A common approach involves the extraction of visual features that can encompass color histograms, texture descriptors, contour shapes and local keypoints. Early works in this domain [21,22,23] applied traditional image processing techniques to extract features such as shape moments and color histograms, leading to initial success in recognizing simple objects. The surge of progress seen in recent years is largely due to the latest developments in deep learning [24], particularly convolutional neural networks (CNNs) and geometric reasoning [25].

Deep learning has had an enormous impact in perception tasks with the design of effective architectures for real-time object recognition, providing significant advancements in accuracy and robustness. CNNs have demonstrated remarkable performance in extracting hierarchical features from images [26]. Transfer learning, where pre-trained models are fine-tuned for specific tasks, has enabled efficient object recognition even with limited training data [27]. A relevant vision-based approach is the one in which the process of recognizing the human-grasped object, across consecutive frames, comprises two sub-processes: hand tracking and object recognition. The hand detection and tracking system is commonly used for defining a bounding box around the grasped object that describes its spatial location. This initial step can, in turn, simplify the object recognition algorithm as it can focus attention solely on the region where the object is likely to be present. This reduces the search space and the required computational resources. Object detection frameworks like YOLO (You Only Look Once) and Faster R-CNN fall under this category. They divide the RGB image into a grid and predict bounding boxes and class probabilities directly from the grid.

In parallel to deep learning, the recent availability of inexpensive RGB-D sensors has enabled significant improvements in scene modeling and human pose estimation. Some studies explore the fusion of multiple modalities to enhance object recognition. These approaches combine visual information with other sensory data, such as depth information from 3D sensors [28,29]. This integration of modalities has shown promise in improving recognition accuracy, especially in scenarios with varying lighting conditions or occlusions. Researchers have also studied how to leverage information from multiple viewpoints (i.e., multi-view 3D object recognition) to enhance recognition accuracy [30]. This approach is particularly relevant for 3D objects, where recognizing an object’s 3D structure from different viewpoints can aid in robust recognition. Techniques like using 3D point clouds, multi-view CNNs or methods that combine RGB images and depth information fall under this category.

Despite their successes, methods within the “Object Sensing” category are often constrained by the variability in object appearances, limited viewpoint coverage and sensitivity to illumination changes. As a result, the focus on object characteristics alone may not provide a complete solution, particularly in situations where the human hand’s interaction with the object plays a crucial role.

### 2.2. Hand Sensing

Recognizing objects based on the interactions of the human hand is a complex problem due to the intricate nature of hand–object interactions (HOIs) and the variability in grasp patterns and gestures [18,31,32,33]. Achieving accurate and real-time recognition involves understanding the relationships and dynamics between a human hand and the objects it interacts with (e.g., the interaction context, the person’s actions and the patterns that emerge over time), as well as the the tactile and kinesthetic feedback generated during manipulation. Additionally, variations in grasp styles, object sizes and orientation further worsen the complexity of the task. Several works propose interaction reasoning networks for modeling spatio-temporal relationships between hands and objects in egocentric video during activities of the daily life, such as playing an instrument, kicking a ball, opening a drawer (one-handed interaction), opening a bottle (two-handed interaction) or cutting a vegetable with a knife. Main advances are due to the development of several human-centric datasets (e.g., V-COCO [34], HICO-DET [31] and HCVRD [35]) that annotate the bounding boxes of each human actor, the object with which he/she is interacting and the corresponding interaction. However, the creation of large-scale, diverse and annotated datasets remains an ongoing effort.

Some works consider the hand–object interaction (HOI) as a manifestation of human intention or purpose of action [36,37,38,39,40]. Despite the growing need for detection and inference of HOIs in practical applications, such as collaborative robotics, the problem of recognizing objects based on hand–object interactions is inherently complex. Instead of addressing the full complexity of HOI recognition, several works have adopted targeted approaches that address specific aspects of the problem without necessarily delving into the entire spectrum of interactions. A recent work investigated the influence of physical properties of objects such as shape, size and weight on forearm electromyiography (EMG) signals and the opportunities that this sensing technology brings in hand–object interaction recognition and/or for object-based activity tracking [41]. Despite the relevance of the work, it is difficult to be applied in collaborative assembly scenarios given the complexity of the required setup that requires sensor attachment, calibration and training. Some other limitations may include user-dependent variability, muscle fatigue and discomfort and/or interference from other electrical devices.

Another line of research, closest to our work, focuses on tracking the positions of hand and finger landmarks during interactions. By monitoring the spatial relationships of these landmarks, these methods aim to deduce the object’s identity based on the specific manipulations applied. This approach captures critical information about the hand’s interaction without necessarily modeling the full complexity of interactions. A glove-based interaction approach has been proposed by Paulson et al. [42] in the HCI domain to investigate a grasp-based selection of objects in office settings. The authors showed that hand posture information alone can be used to recognize various activities in an office, such dialing a number, holding a mug, typing at the keyboard or handling the mouse. The classification of hand posture is performed using the nearest-neighbor algorithm. In a similar work based on a data glove, Vatavu et al. [43] proposed the automatic recognition of the size and shape of objects using the posture of the hand during prehension. The objects used in the experiments consisted of six basic shapes (cube, parallelepiped, cylinder, sphere, pyramid and a thin plate) and, for each shape, three different sizes (small, medium and large). Twelve right-handed participants took part in the experiments using a 5DT Data Glove Ultra equipped with 14 optical sensors. These sensors were distributed as follows: 10 sensors measure finger flexion (two sensors per finger) and four sensors measure abduction between fingers.

The study compared several classifiers derived from the nearest-neighbour approach with a multi-layer perceptron (MLP) and a multi-class support vector machine (SVM). The best results were achieved with the *K*-nearest-neighbor classification approach when combining the results of individual postures across an entire time window of half a second. The experiments carried out included the capture of hand postures when grasping and maintaining a stable grip for a reliable translation of the objects. The results show that object size and shape can be recognized with up to 98% accuracy when using user-specific metrics. The authors also pointed out the lower accuracy for user-independent training and the variability in the individual grasping postures during object exploration. Although in general the proposed approach recognizes the physical properties of the grasped objects with high accuracy, wearing a glove directly on the hand is intrusive and troublesome, interfering with the natural movement of the fingers.

When attempting to model human grasping, researchers have focused their attention on defining a comprehensive taxonomy of human grasp types [44] and the multifaceted factors that influence the choice of grasping, including user intentions [45], object properties [46] and environmental constraints [47]. Mackenzie and Iberall [45] theorize the existence of a cognitive model that converts the object’s geometry properties and user’s intent into a motor program driving the hand and finger motions. From this seminal work, several studies on human reach-to-grasp actions have consistently shown that the natural kinematics of prehension allows for predicting the object he/she is going to grasp, as well as the subsequent actions that will be carried out with that object. Feix et al. [46] provided an analysis of human grasping behaviors showing the correlation between the properties of the objects and the grasp choice. More recently, the works of Betti et al. [48] and Egmose and Koppe [49] focus on the reach-to-grasp phase. Their finding shows that grasp formation is highly correlated with the size and shape of the object to be grasped, as well as strongly related to the intended action. These insights promise improved interaction by exploring the ability with in which the robot can predict the object the user intends to grasp or to recognize the one he/she is already holding, provided that the hand kinematics information is extracted and processed in real time.

In line with this, Valkov et al. [50] investigated the feasibility and accuracy of recognizing objects based on hand kinematics and long short-term memory (LSTM) networks. The data are extracted from a Polhemus Viper16 electromagnetic tracking system with 12 sensors attached to the hand and fingers. On the one hand, the study focuses on the size discrimination of nine synthetic objects: three regular solids (sphere, box and cylinder) in three different sizes (small—2 cm, medium—4 cm and large—6 cm). On the other hand, a different set of seven objects (pen, glue, bottle, Rubik’s cube, volcano-egg, toy and scissor) was used for object discrimination. The data recorded during the experiments include a phase in which participants were asked to reach and grasp the object starting from a fixed initial position. The results demonstrated that LSTM networks can predict the time point at which the user grasps an object with 23 ms precision and the current distance to it with a precision better than 1 cm. Furthermore, the size and the object discrimination during the reach-to-grasp actions were achieved successfully with an accuracy above 90% using *K*-fold cross-validation. Although the results are still preliminary, the leave-one-out cross-validation showed a significant degradation in the performance of the models compared to the *K*-fold validation. While the tracking system offers many advantages, there are also practical limitations such as sensor attachment and comfort, line-of-sight requirements, interference and noise as well as calibration and drift.

The solution adopted in our paper focuses on detecting and tracking the hand and finger keypoints from visual data. The proposed framework combines the strengths of Mediapipe in detecting hand landmarks in an RGB image with a deep multi-class classifier that predicts the grasped object from a set of 21 keypoints. Accordingly, our object recognition system operates based on different principles, including the sensing device, the tracking method and the machine learning approaches. From the point of view of application in industrial settings, the proposed system has two strengths when compared to the use of data gloves or electromagnetic motion capture systems. First, the simplicity of installation is associated to a much less complex and costly setup. Second, the non-intrusiveness of the required setup is a valuable factor to accelerate the acceptance of these technologies by humans in carrying out collaborative tasks (a process also referred to as “user adoption”). In contrast, vision-based hand tracking is affected by occlusions, changes in light conditions and cluttered backgrounds. Furthermore, these problems are difficult to overcome with deep-learning techniques given the data dependency and generalization problems against hands, objects and lighting conditions outside the training sets. Overall, this paper contributes to advances in understanding the opportunities and limitations of using this novel approach for the recognition of human-grasped objects.

## 3. Materials and Methods

**Figure 1:**
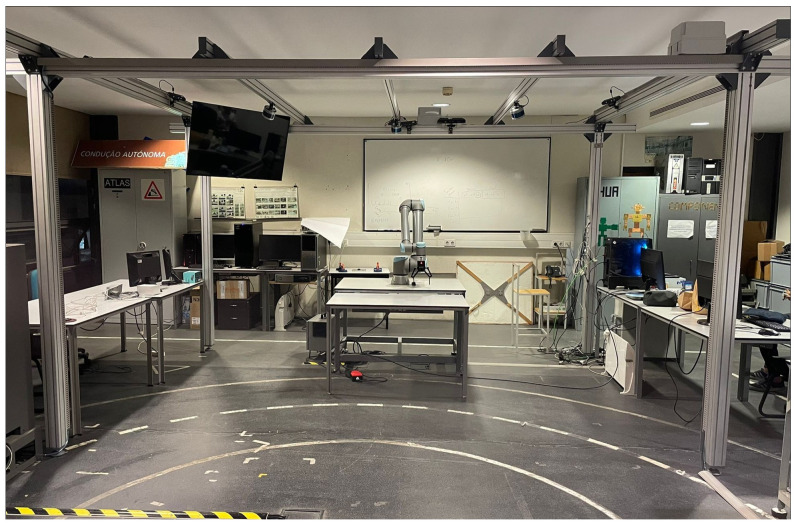
The prototype collaborative cell LARCC.

The work described in this paper is part of the AUGMANITY project (https://www.augmanity.pt, accessed on 31 October 2023) that aims to develop technologies to support people operating in industrial environments. The experimental setup comprises the integration of both hardware and software components in a prototype collaborative cell (LARCC), as illustrated in Figure 1. The LARCC is equipped with a UR10e collaborative robot and multimodal sensor devices, including three LiDAR Velodyne sensors and four Orbbec 3D cameras distributed throughout the work volume. The software architecture is built upon the Robot Operating System (ROS) middleware [51], providing a robust framework for communication and coordination among the various components. In this context, this section provides a description of the materials used during this study and the methodological approaches followed to face the key challenges.

### 3.1. Proposed Approach

**Figure 2:**
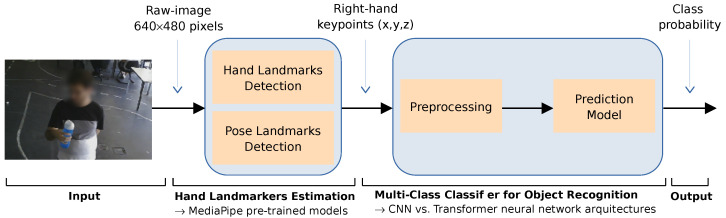
The proposed learning-based framework combines the MediaPipe pre-trained models for hand/object detection and tracking with a multi-class classifier for object recognition based on the hand keypoints.

We propose a learning-based framework to enable an assistive robot to recognize the object grasped by the human operator. As illustrated in Figure 2, the framework combines the strengths of MediaPipe in detecting hand landmarks in an RGB image with a deep multi-class classifier that predicts the object based on the configuration of the user’s hand after grasping it. MediaPipe consists of a set of libraries and tools commonly used in machine learning pipelines for advanced real-time vision-based applications [19]. The Hand Landmark Detection model [52] uses two sub-modules: a hand palm detection model and a hand landmark model. Each frame of an RGB input is fed into the palm detection model, which produces a bounding box based on the palm. The hand landmark model uses this bounding box and returns the keypoint localization of 21 landmarks, including the fingertips, the finger joints (knuckles) and the base of the palm.

The output of the pre-trained model provides the (x,y,z) coordinates of landmarks for each detected hand. The (x,y) coordinates represent the horizontal and vertical positions of the landmark on the image plane, while the *z*-coordinate represents an estimate of the relative depth with respect to the wrist reference [53]. Our work focuses on tracking the right hand by combining the Hand Landmark detection and the Pose Landmark Detection pre-trained models. This strategy proved to be useful in order to enhance the reliability of the process of extracting the coordinates of the right-hand keypoints from each frame.

The multi-class classifier for object recognition faces several challenges. First, we have limited information about the three-dimensional configuration of the hand, namely whether the hand configurations involve overlapping fingers or positions close to each other in the image plane. Consequently, the *z*-coordinate (relative depth) revealed to be a critical element for discriminating complex hand configurations. Second, the coordinates provided by MediaPipe can vary in scale and rotation depending on the hand’s distance from the camera and the hand’s orientation in the image, adding complexity to the task. For these reasons, a deep learning model able to learn complex features directly from the MediaPipe coordinates will be explored and evaluated with a view to its generalization ability in different scenarios and for various users.

The learning problem involves a mapping between two spaces f(X,θ):X→Y, where $X\in R^{3\times 21}$ is the set of possible spatial coordinates of the hand keypoints, $Y\in R^{M}$ is the set of possible *M* output classes and θ the model parameters. Let $D=\{\left(x_{1},y_{1}\right),\cdots ,\left(x_{n},y_{n}\right)\}$ be a training dataset of *n* examples where $x_{i}\in X$ is an input and $y_{i}\in Y$ is the corresponding ground truth class label. Given a new instance $x_{new}$, the task is to predict its corresponding object class $y_{pred}$, such that

(1) $$y_{pred}=f\left(x_{new}\right).$$

The model aims to minimize a chosen loss function *L* that quantifies the dissimilarity between the predicted class $y_{pred}$ and the ground truth class $y_{i}$. Formally, the training process seeks to find the optimal parameters $\hat{\theta }$ of the mapping function *f* by solving the following optimization problem:

(2) $$\hat{\theta }=\underset{\theta }{\mathrm{argmin}}\frac{1}{n}\sum_{i=1}^{n}L\left(f_{\theta }\left(x_{i}\right),y_{i}\right),$$

where $f_{\theta }\left(x_{i}\right)$ is the predicted class label for sample $x_{i}$ using the model with parameters θ, and *L* is a suitable loss function. Upon successful training, the model *f* can be used for predicting the object class $y_{pred}$ for new instances of hand keypoints.

### 3.2. Data Acquisition

Orbbec Astra Pro cameras were used in the project setup to facilitate accurate perception and real-time awareness of the collaborative environment. The camera data are integrated into the perception pipeline to provide depth sensing capabilities, allowing the robotic system to perceive and understand its environment in three dimensions. In this study, we restrict ourselves to using the integrated RGB color camera installed at the top of the cell structure (at a height of 2.30 m) to cover the working space. The MediaPipe software was used to extract the required landmarks automatically from the video input.

A recent study by Amprimo et al. [53] evaluated the performance of the basic MediaPipe Hand [52] and an enhanced solution using depth data [54] against a motion capture system using an Optitrack solution comprising six Prime13 cameras. The focus was on the usage of such hand-tracking framework in clinical applications, such as automatic motion quality assessment, as well as the influence on the tracking quality of factors such as distance from the camera, camera viewing angle and velocity of the motion. The results show that the use of the hands model based on an RGB input provides a good level of trust in terms of tracking accuracy.

**Figure 3:**
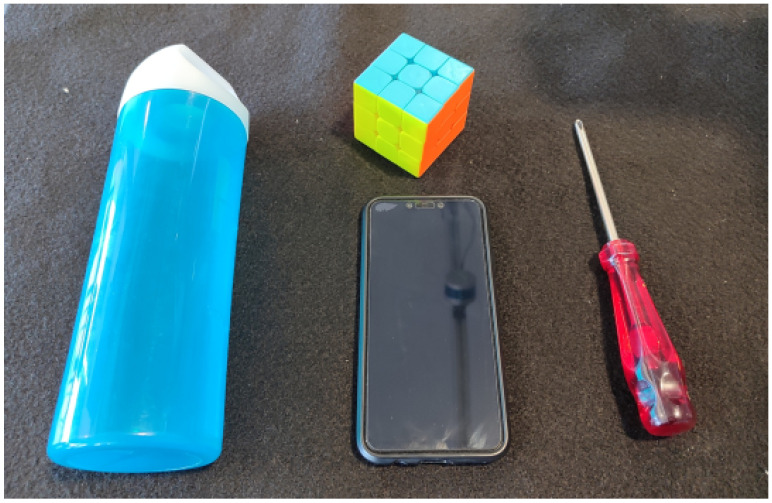
The objects used in the study include a water bottle, a Rubik’s cube, a smartphone and a screwdriver.

In the same line of thought, the question arose of evaluating the reliability of the hand-tracking software in situations where the hand grasps an object, taking into account the model’s level of confidence [52] in the localization of the hand landmarks. The four objects selected for this study are all “graspable”, i.e., more or less rigid. They include a cylindrical water bottle, a Rubik’s cube, a flat and thick smartphone and a small and sharp screwdriver (Figure 3). Given the differences in shape, size and/or weight, we are interested in discriminating these four objects based on the configuration adopted by the hand while interacting with them.

Thus, before acquiring the dataset, we evaluated the success of the MediaPipe framework in identifying the hand keypoints in two cases. First, the hand remained stationary during the acquisition of 250 frames, considering both the hand without interaction with an object and the hand grasping each of the objects selected in the study. The results show that MediaPipe achieves a success rate of nearly 100% when the hand is not interacting with an object and fluctuates between 93.2% and 99.2% when it grasps an object. Second, the hand performed random movements, resulting in lower success rates: approximately 98.4% without an object and values between 83.2% and 84.8%, depending on the object. These success rates can be considered acceptable since, once in operation, the classifier will tend to consider several frames, and not just one, to make a more reliable decision. Despite this, we were able to observe more complex occlusion situations in which MediaPipe did not return valid coordinates for a 4 s interval. This may lead to rethinking the best camera location (e.g., environment- versus robot-mounted camera) and, eventually, the number of cameras to use.

This study involved the participation of three right-handed male volunteers aged between 23 and 26 years old. Participants were asked to naturally grab and hold an object placed on a table, followed by executing small movements of the hand in free space. These movements were performed while introducing random variations in the hand’s orientation relative to the RGB camera to ensure diversity in the points of view from which the hand–object interaction was observed. However, the successive frames led to similar grasping patterns from different views. In order to investigate intra-user variability and to ensure robust model training, users were instructed to perform multiple grasping trials of the selected object across four distinct acquisition sessions.

Bearing this in mind, the data acquisition system was designed to facilitate the fast generation of training datasets, accommodating the inclusion of new users and/or additional acquisition sessions. On the one hand, the system is integrated into the workflow of the proposed object recognition framework. On the other hand, it is particularly well-suited for implementation in industrial settings where end-users may not possess extensive expertise in machine learning or computer vision. The instructions provided to users during the data acquisition sessions were intentionally straightforward, ensuring that non-experts can readily participate in the process.

Videos over four sessions per user were recorded at 10 frames per second. For each object and each user, four data acquisition sessions were carried out, which gave rise to the dataset used in this study. Therefore, the dataset consists of a total of 11,054 samples, distributed practically equally across the three participants (around 3600 samples per participant) and the four objects (between 2618 and 2849 samples per object). The exact number of samples of the entire dataset per class and per user is shown in Table 1. In order to properly evaluate the classifier for object recognition, we used this new dataset which is available online.

### 3.3. Data Pre-Processing

The pre-processing steps applied to the raw data in preparation for a reliable dataset include data cleaning, data transformation and data normalization. Data cleaning involved identifying and addressing inconsistent values in those situations where MediaPipe do not provide valid keypoint coordinates according to the specified confidence parameters. All these outlier samples are removed automatically from the dataset. The next step was to apply a transformation of the original coordinates of the keypoints (raw data), which are already normalized within the range from 0 to 1, into coordinates relative to a reference. Specifically, for each keypoint P=(x,y,z), we subtract the coordinates of the reference point $P_{ref}=\left(x_{ref},y_{ref},z_{ref}\right)$ from them to obtain relative coordinates $P_{rel}=\left(x_{rel},y_{rel},z_{rel}\right)$. In this study, the reference is defined as the centroid *C* of the set of hand keypoints. This transformation into relative coordinates is particularly useful because the absolute position of the hands in the image may vary from frame to frame due to different distances from the camera or hand orientations. Instead, relative coordinates are translation-invariant and they reduce the influence of any rotations that may be present in the raw data. Therefore, the network will focus on the spatial relationships between keypoints, rather than their absolute positions, making it less sensitive to hand orientations and scale variations.

**Figure 4:**
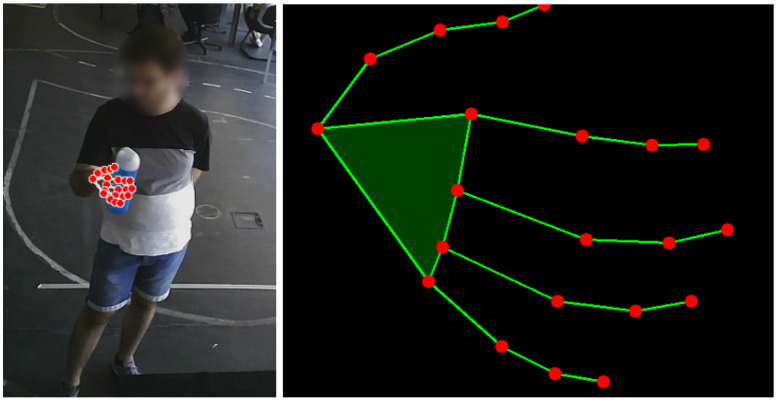
The keypoints projected in the image (**left**) and the normalized representation (**right**).

After obtaining the relative coordinates with respect to the reference point, we apply scaling to each dimension independently by dividing by an appropriate constant to ensure that the hand’s representation spans the entire range, as follows:

(3) $$scaleFactor=\frac{0.5}{\max(\left\{\left|x_{i}\right|,\left|y_{i}\right|,\left|z_{i}\right|\right\}:i=1,\cdots ,n)}\;,$$

where $\left\{x_{i},y_{i},z_{i}\right\}$ denote relative coordinates. This feature scaling revealed to be a valuable pre-processing step to help make the data more consistent, helping the model to learn the relevant patterns without being influenced by variations in hand’s position, hand’s size or scale. Furthermore, it helps to maximize the separation among keypoints, helping the model to discriminate the output class. Finally, a uniform adjustment is made by adding 0.5 to each coordinate, centering the points between 0 and 1 on the scale. Figure 4 shows an example of the keypoints projected in the image plane and the respective normalized representation expressed according the previous steps, that is

(4) $$P_{norm}=\left(P-C\right)\times scaleFactor+0.5\;.$$

Before describing the methodology adopted for recognizing grasping patterns, we conducted a statistical analysis on the entire dataset obtained from MediaPipe using K-means clustering. After applying pre-processing steps to the raw data, we divided the dataset, consisting of 11,054 samples, into training (80%) and testing subsets (20%). Initially, the K-means algorithm was applied to learn the cluster structure within the training data. Subsequently, the model’s performance was assessed using a separate and previously unseen test dataset.

**Figure 5:**
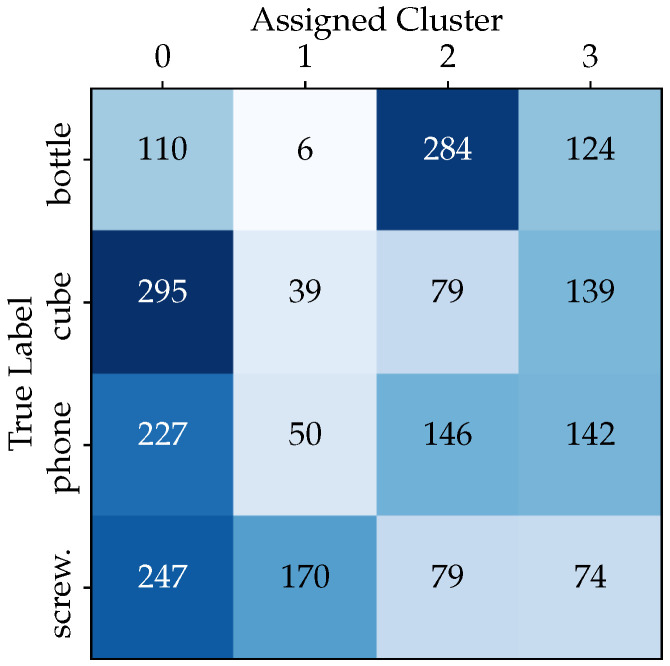
The distribution of test dataset samples from each class within each cluster.

The analysis results are visualized in a 4 × 4 matrix, denoted as the “True Labels” vs. “Assigned Cluster” matrix (see Figure 5). This matrix provides a comprehensive view of the K-means clustering method’s performance in object recognition and it enables the computation of the proportion of objects assigned to each cluster. This cluster analysis provides valuable insights into the relationships between hand poses and class labels. First, the analysis of the relationship between clusters and class labels in hand pose data reveals that certain clusters, like Clusters 0 and 3, exhibit a diverse mix of all classes. Second, in the test data, Cluster 0 encompasses a substantial 39.8% of the samples. Third, and of utmost importance, the K-means clustering results indicate that the clusters do not closely align with the class labels, which may indeed present challenges for the chosen classifier.

The complexity of the problem is increased by user dependency and the variability observed within the same subject during different trials of grasping the same object. Given these challenges, a supervised learning approach, such as a CNN, emerges as a suitable choice, as it can autonomously learn hierarchical features and patterns directly from the data, irrespective of the initial cluster structure. Deep learning models, in particular, excel in addressing the intricacies of hand posture recognition and demonstrating robust generalization across different users, effectively capturing intra-user variations, provided that the training dataset exhibits diversity and represents the target scenarios.

### 3.4. Neural Network Architectures

This subsection outlines the methodology employed for recognizing and classifying objects held in the user’s hand using extracted hand and finger keypoints from MediaPipe. The goal is to distinguish between four different objects based on the hand configuration. To achieve this, the performance of two distinct deep learning architectures will be compared: a convolutional neural network (CNN) and a transformer. The selection of the transformer was driven by its strengths in handling spatial features and capturing dependencies specific to the object recognition problem. Unlike CNNs, which typically employ local receptive fields, transformers can efficiently capture global context information. In particular, the self-attention mechanism is expected to capture these dependencies by assigning different weights to keypoints based on their relative positions. This enables the model to focus on keypoint interactions that are most relevant for discriminating the different hand postures.

The deep models were developed from scratch, aiming to achieve an enhanced performance in the specific problem domain using the same dataset. The CNN model architecture was designed paying attention to the choice of convolutional layers, their kernel sizes and the structure of fully connected layers. The keypoints were structured in a two-dimensional 3 × 21 matrix representation, with each of them possessing positional significance. In order to enhance the model’s performance, we conducted an extensive hyperparameter search involving the learning rate, dropout rate, kernel size and the number of convolutional and dense layers. We employed a four-fold cross-validation strategy to evaluate different combinations of hyperparameters.

The backbone of our CNN comprises a total of three convolutional layers each with 64 feature maps and ReLU activation functions. The first layer uses a kernel size of 3 × 3 pixels performing a 1D convolution on the 3 × 21 data with a stride of 1 pixel. The flattened output from the final layer is connected to a dense layer with 128 neurons, followed by another dense layer with the number of neurons equal to the number of classes. The output layer consists of the final connect layer with softmax activation. The softmax function takes a vector of real-valued scores (often called logits) and transforms them into a probability distribution over multiple classes. For our classification task with 4 classes, the output layer has 4 neurons, each representing the probability of the input belonging to a particular class. To prevent overfitting, we incorporate dropout layers with a dropout rate of 0.5 after each fully connected layer. We adopted a learning rate of 0.001, a batch size of 128 and early stopping. We trained our CNN using the Adam optimizer with a initial learning rate of 0.001, optimizing the categorical cross-entropy loss function and initializing weights with He initialization to ensure efficient convergence and effective multi-class image classification.

In what concerns the transformer model, a careful selection of hyperparameters was carried out to excel in the task of hand pose classification, giving rise to two layers for the encoder stack and multi-head self-attention with four heads to capture relationships among keypoints. The hand keypoints are treated as a sequence of feature vectors that feed the model for classification. The encoder stack consists of multiple identical layers, each housing the following components: multi-head self-attention, layer normalization and feedfoward neural networks. Within each layer, multi-head self-attention is applied to capture dependencies among the keypoints. We use four attention heads for enhanced feature extraction. Following self-attention, two position-wise feedforward neural networks are employed to process the attended features and capture complex patterns. Layer normalization is applied after each sub-layer to stabilize the activations and facilitate training convergence. During training, a learning rate of 0.0001 was employed, with a gradual decay schedule to facilitate convergence. The model was optimized using the Adam optimizer and the categorical cross-entropy loss. Training was conducted with a batch size of 64 sequences, balancing training efficiency and GPU memory utilization.

## 4. Results

This section provides a comparative analysis of the performance of a CNN model against a transformer model for the recognition of the human-grasped object from the hand keypoints. We designed three main experiments and evaluated the classification performance of the two architectures under comparison when they are fed with the same data. With the three experiments, we intend to study the generalization capacity of the deep model in unseen data, taking into account the different users and the different data acquisition sessions. The algorithms were implemented in Python language, using the Keras library. The computer-intensive training phase is supported by a deep learning server equipped with an AMD Ryzen Threadripper 2950X CPU running at 2.50 GHz, 4 NVIDIA GeForce RTX 2080 Ti and 128 GB RAM.

### 4.1. Experiments and Metrics

In the context of our research on developing a hand–object recognition classifier utilizing keypoints provided by the MediaPipe Hands model, we conducted a series of experiments to evaluate the classifier’s performance comprehensively, as follows:

- Experiment 1: Session-Based Test. The first experiment aims to assess the impact of session-based testing on the classifier’s performance. For that purpose, the classifier will be trained on data from all users and all acquisition sessions except one that will be used for testing.
- Experiment 2: User-Specific Test. In our pursuit of refining the hand–object recognition classifier, we will conduct a second experiment with a focus on individual user data. This experiment aims to provide insights into how the classifier performs when trained and tested on data collected from a single user, with the process repeated separately for each of the selected users.
- Experiment 3: Leave-One-User-Out Test. The third experiment follows a distinctive approach termed the “Leave-One-User-Out Test”. This experiment is designed to evaluate the classifier’s performance when trained on data from two users and tested on data from the third user.

The entire dataset is initially split into training and test sets using an indicative 80–20 ratio (the exact ratio can vary slightly depending on the experiment). Subsequently, the training set is further divided into a training subset and a validation subset using the same ratio. All experiments carried out take into account the intrinsic variability in performance estimation by conducting 50 Monte Carlo simulations.

Confusion matrices are used to help identify specific areas where the model may excel or struggle in recognizing the grasped object. Additionally, metrics such as accuracy, precision, recall and *F*1-score are evaluated by computing the four counts that constitute a confusion matrix: the number of correctly predicted class examples (True Positives, TP), the number of correctly predicted examples that do not belong to the class (True Negatives, TN), and examples that either were incorrectly assigned to the class (False Positives, FP) or that were not predicted as class examples (False Negatives, FN).

The accuracy was defined as the proportion of correctly classified examples among all samples in the dataset, calculated as follows:

(5) $$ACC=\frac{TP+TN}{TP+TN+FP+FN}\;.$$

Precision is used to quantify the ability of the model to correctly identify positive examples among all examples predicted as positive,

(6) $$P=\frac{TP}{TP+FP}\;.$$

Recall measures the proportion of actual positive examples correctly predicted by the model. It was calculated as follows:

(7) $$R=\frac{TP}{TP+FN}\;.$$

The *F*1-score is also an one-dimensional indicator providing a balance between precision and recall,

(8) $$F1=\frac{2\times P\times R}{P+R}\;.$$

### 4.2. Session-Based Testing

**Figure 6:**
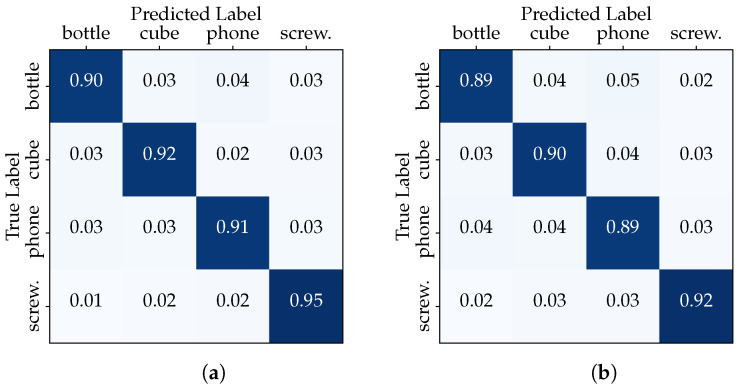
“Full Dataset” confusion matrices: (**a**) CNN model, (**b**) transformer model.

In order to investigate the influence of session-based testing, we divided the dataset into two configurations. In the first configuration, referred to as the “Full Dataset” scenario, we employed the entire dataset consisting of 11,054 samples for both training and testing. First, the dataset was randomly split into training (6632 samples), validation (2211 samples) and testing (2211 samples) subsets. This setup aims to evaluate the classifier’s performance when trained on a diverse set of hand–object interactions. Table 2 summarizes the results of evaluating the performance of the two models in the “Full Dataset” scenario in terms of accuracy, precision, recall and F1-score. The CNN and transformer show similar results with robust scores around 92%. The confusion matrices in Figure 6 show that the classifiers excel at distinguishing between the different classes, maintaining high accuracy.

**Figure 7:**
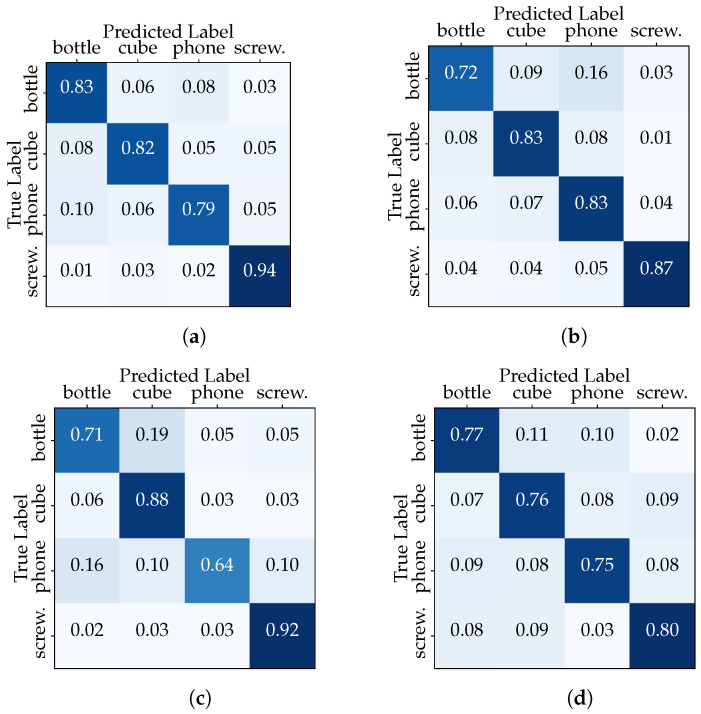
“Session-Based Testing” confusion matrices (CNN model): (**a**) Session 1, (**b**) Session 2, (**c**) Session 3, and (**d**) Session 4.

This outcome underscores the classifier’s ability to generalize across a diverse range of hand–object interactions, as it was trained on a dataset encompassing multiple users and multiple data acquisition sessions. In the second configuration, we isolated one session from each user for testing (2731 samples), while the remaining sessions from all users were used for training (8289 samples). We aim to determine how the inclusion of session-specific data impacts the classifier performance. The results depicted in Table 3 show the discrepancy in classifier accuracy between the “Full Dataset” and the “Session-Based Testing” scenarios. Furthermore, the confusion matrix in Figure 7 compares the actual target with those predicted by the CNN model. The classifier is making accurate and correct predictions for the majority of classes, while it is struggling to accurately recognize examples belonging to specific classes, such as the “phone” in the third data acquisition session (the same with the transformer).

### 4.3. User-Specific Test

**Figure 8:**
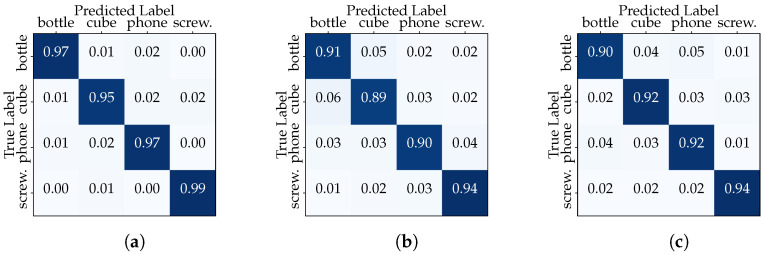
“Full User Dataset” confusion matrices (CNN model): (**a**) User1, (**b**) User2 and (**c**) User3.

The experiment described in this subsection focuses on the variability in hand configurations and keypoint patterns for the same user over time (i.e., under different conditions imposed by the four data acquisition sessions carried out). Once again, we constructed two distinct dataset configurations for each user. In the first configuration (“Full User Dataset”), we used the entire dataset for a single user, with the process repeated separately for all others. This simulates a scenario where the classifier is trained and tested on all available data for a single user based on a random split using a 80–20 ratio. The performance of the CNN and transformer models in the “Full User Dataset” scenario is summarized in Table 4. The CNN network presents slightly better results than the transformer in different metrics, while User1’s performance stands out. The confusion matrices show that the classifiers excel in distinguish between the different classes with high accuracy (see Figure 8 relating to the CNN model).

**Figure 9:**
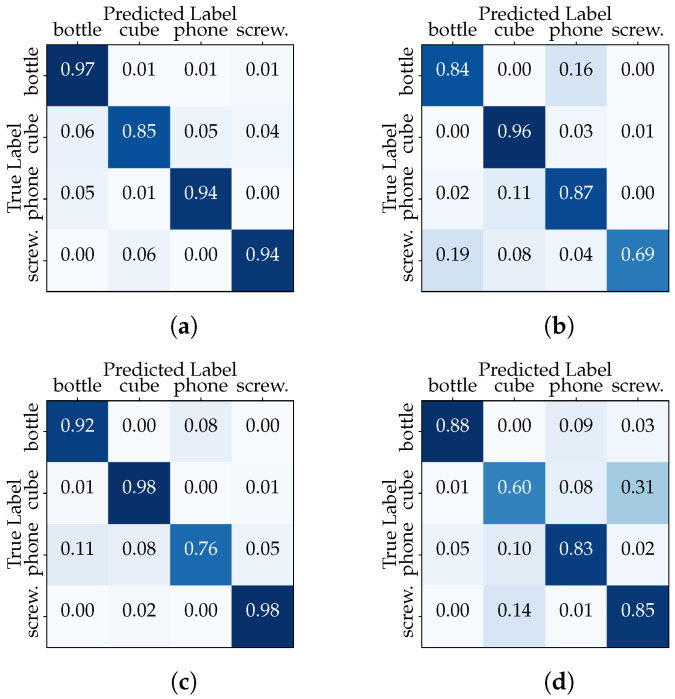
“Session-Based User1 Testing” confusion matrices (CNN model): (**a**) Session 1, (**b**) Session 2, (**c**) Session 3, and (**d**) Session 4.

In the second configuration, we isolated the data of one acquisition session for a specific user, dedicating them exclusively to the testing phase. Meanwhile, the remaining sessions’ data from the same user were employed for training. This “Session-Based User Testing” setup mirrors scenarios where a classifier must adapt to recognize objects manipulated by a user based on a limited history of interactions. The results in Table 5, relating to User1, reveal interesting insights into the classifier’s adaptability within the context of different user behaviors across multiple sessions. First, we note varying levels in all metrics across sessions in the range between 78.8% and 92.6%. The decrease in the evaluation metrics between the “Full User Dataset” and “Session-Based User Testing” scenarios highlights the importance of user-specific adaptation. The confusion matrices (see Figure 9) also reveal lower performances in certain classes, but these vary from session to session. These results emphasize the importance of considering session-specific variations and user behaviors when training and evaluating the classifier.

### 4.4. Leave-One-User-Out Test

The third experiment follows a distinctive approach termed the “Leave-One-User-Out Test”. This is particularly relevant in scenarios where the data are collected from multiple users (i.e., dealing with user-dependent data), and the goal is to evaluate the model’s generalization ability to new individuals who were not part of the training data. In this case, the process involves training the model on data from all users except one (the user to be left out), and then evaluating the model’s performance on the data from the left-out user. This experiment allows for the observation of a trend and it showcases the challenges of generalization to unseen users. In order to identify the trend, the process is repeated for each user, training the model on all users except the one being evaluated. This approach ensures that each user’s data are used once as a test set (around 3660 samples), while the remaining data are employed for training (around 7350 samples).

Table 6 shows the results obtained considering that only one user’s data (all sessions) are used in testing the model. Although the classifier recognizes to some extent objects grasped by “User1” when it has not been explicitly trained on it, the lower performance for “User2” and “User3” indicates limitations in generalization to users with distinct grasping patterns. The results indicate the difficulties the deep model faces when adapting to a new user in the absence of a dedicated training period. This emphasizes the need for personalized models or strategies that can adapt to individual user behaviors. In real-world scenarios, users may exhibit diverse hand–object interaction patterns and models should be capable of accommodating these variations. Whatever strategy is adopted, ensuring diverse and representative data (e.g., a bigger dataset) will be crucial for improving the results, either using a convolutional network as a transformer.

### 4.5. Discussion

The experiments carried out provide valuable insights into the classifier’s performance in different testing scenarios, especially for real-world applications. The results of the “User-Specifc Test” show that the classifier’s performance is influenced by the variability in interaction patterns across different sessions. This emphasizes the importance of considering session-specific variations when training and evaluating the classifier in user-centric applications (e.g., single-operator workstations). The lower accuracy in certain sessions and classes suggests the need for model refinements to address changes in data distribution between different work sessions. Pre-training a model on a large and diverse dataset and then fine-tuning it using data from a new session can be effective. Likewise, monitoring the model’s performance in real time and periodically retraining it with new and diverse data can help the model adapt to changing conditions and variations in different work sessions.

In dynamic settings where multiple users may interact with objects differently across multiple sessions, it is crucial for the classifier to adapt and maintain performance. The declining trend observed in the “Session-Based Testing” scenario suggests that the classifier may have difficulties in recognizing grasping patterns effectively when faced with new sessions that deviate from those in training. Once again, these results highlight the importance of considering session-specific variations when acquiring the dataset. From the perspective of model development in practical applications, the findings of this experiment emphasize the need for ongoing model refinements and adaptation strategies. Techniques such as session-specific fine-tuning or the incorporation of session-related features may prove valuable in enhancing the classifier’s performance in real-world, dynamic environments.

The findings from the “Leave-One-User-Out Test” highlight the importance of personalized modeling approaches to account for user-specific patterns in the hand–object recognition system. In order to enhance the classifier’s performance, it may be necessary to consider user-specific fine-tuning that can help the model better capture the nuances of new users. This personalized training can lead to better convergence and performance. This reflects the importance of actively collecting data from new users in a systematic way to adapt the model. In line with this, we can foresee the implementation of a system that allows for continuous model updating as new user data becomes available, i.e., the model can adapt and improve over time.

## 5. Conclusions

This paper proposed a novel DL-based framework for hand–object recognition using the MediaPipe Hands model and a multi-class classifier that predicts the grasped object from the hand keypoints. This study was focused on the classifier’s generalization ability, remarking the importance of carrying out an effective evaluation before the system is applied in real-world scenarios. Throughout the experiments, we observed variations in performance, particularly in scenarios involving session-based testing and user-specific adaptation. The main results emphasize the importance of continuous model monitoring, retraining and active data collection to enable the classifier to generalize effectively across diverse user behaviors and grasping patterns. Personalized modeling approaches and fine-tuning strategies may be useful to address the challenges at hand. In this context, careful consideration of dataset dimensionality is essential to optimize model performance and obtain meaningful insights from the data.

Our findings offer valuable insights into the factors influencing the performance of the classifier and the implications for real-world applications. As we move forward, future research will explore advanced techniques to further enhance the adaptability and generalization capabilities of the hand–object recognition system, namely by exploring data from human-grasping databases like [55]. This work sets the stage for ongoing improvements with the ultimate goal of delivering effective solutions for a wide range of applications.

## Figures and Tables

**Table 1:** Number of samples of the entire dataset per class and user.

Dataset	Bottle	Cube	Phone	Screw	Total
**User1**	828	928	950	957	3663
**User2**	886	926	939	946	3697
**User3**	904	907	937	946	3694
**Total**	2618	2761	2826	2849	11,054

**Table 2:** “Full Dataset” performance metrics.

Model	Accuracy	Precision	Recall	F1-Score
CNN	**0.9210**	**0.9214**	**0.9211**	**0.9211**
Transformer	0.9017	0.9020	0.9016	0.9017

**Table 3:** “Session-Based Testing” performance metrics where data from each session only appear in one set. For example, the “Session 1” column means that data from that session of all users are used in testing, while the remaining sessions are used for training.

Metric	Model	Session 1	Session 2	Session 3	Session 4
Accuracy	CNN	**0.8493**	**0.8138**	0.7844	**0.7718**
	Transformer	0.8458	0.8027	**0.7902**	0.7613
Precision	CNN	**0.8515**	**0.8160**	0.8024	**0.7723**
	Transformer	0.8469	0.8028	**0.8045**	0.7623
Recall	CNN	**0.8493**	**0.8138**	0.7844	**0.7718**
	Transformer	0.8458	0.8027	**0.7902**	0.7613
F1-Score	CNN	**0.8499**	**0.8136**	0.7878	**0.7717**
	Transformer	0.8461	0.8019	**0.7932**	0.7614

**Table 4:** “Full User Dataset” performance metrics.

Metric	Model	User1	User2	User3
Accuracy	CNN	**0.9674**	**0.9100**	**0.9163**
	Transformer	0.9423	0.8929	0.8730
Precision	CNN	**0.9678**	**0.9109**	**0.9171**
	Transformer	0.9435	0.8943	0.8745
Recall	CNN	**0.9674**	**0.9100**	**0.9163**
	Transformer	0.9423	0.8929	0.8730
F1-Score	CNN	**0.9675**	**0.9101**	**0.9164**
	Transformer	0.9423	0.8930	0.8729

**Table 5:** “Session-Based User1 Testing” performance metrics (each column indicates the specific session used in testing the model).

Metric	Model	Session 1	Session 2	Session 3	Session 4
Accuracy	CNN	**0.9257**	**0.8364**	**0.9053**	**0.7883**
	Transformer	0.9078	0.8171	0.8742	0.7636
Precision	CNN	**0.9288**	**0.8543**	**0.9135**	**0.7971**
	Transformer	0.9112	0.8401	0.8806	0.7846
Recall	CNN	**0.9257**	**0.8364**	**0.9053**	**0.7884**
	Transformer	0.9078	0.8172	0.8742	0.7636
F1-Score	CNN	**0.9261**	**0.8382**	**0.9073**	**0.7892**
	Transformer	0.9083	0.8196	0.8759	0.7671

**Table 6:** “Leave-One-User-Out Test” performance metrics where data from each user only appear in the test set. For example, the “User1” column means that data from that user are used in testing, while the data from the remaining users are used for training.

Metric	Model	User1	User2	User3
Accuracy	CNN	0.7969	**0.5827**	**0.5488**
	Transformer	**0.8006**	0.5730	0.5350
Precision	CNN	**0.8123**	**0.5889**	**0.5675**
	Transformer	0.8094	0.5794	0.5492
Recall	CNN	0.7969	**0.5827**	**0.5488**
	Transformer	**0.8006**	0.5730	0.5350
F1-Score	CNN	0.8008	**0.5791**	**0.5483**
	Transformer	**0.8028**	0.5702	0.5321

## Data Availability

Publicly available datasets were analyzed in this study. This data can be found here: https://www.kaggle.com/datasets/pedromiglou/human-grasping-patterns-for-object-recognition, accessed on 31 October 2023.
